# Culture-dependent and universal constructs and promoting factors for the process of personal recovery in users of mental health services: qualitative findings from Japan

**DOI:** 10.1186/s12888-022-03750-4

**Published:** 2022-02-10

**Authors:** Akiko Kanehara, Haruna Koike, Yumiko Fujieda, Sayaka Yajima, Asami Kabumoto, Yousuke Kumakura, Kentaro Morita, Yuki Miyamoto, Masahiro Nochi, Kiyoto Kasai

**Affiliations:** 1 Department of Neuropsychiatry, University of Tokyo Hospital, 7-3-1 Hongo, Bunkyo-ku, Tokyo, 113-8655 Japan; 2 Department of Pathology of Mental Diseases, National Institute of Mental Health, National Center of Neurology and Psychiatry, Tokyo, Japan; 3 Department of Rehabilitation, University of Tokyo Hospital, Tokyo, Japan; 4 Department of Mental Health/ Psychiatric Nursing, Graduate School of Medicine, University of Tokyo, Tokyo, Japan; 5 Department of Psychiatric Nursing, Graduate School of Medicine, University of Tokyo, Tokyo, Japan; 6 Department of Clinical Psychology, University of Tokyo, Tokyo, Japan; 7 Department of Neuropsychiatry, Graduate School of Medicine, The University of Tokyo, Tokyo, Japan; 8 The International Research Center for Neurointelligence (WPI-IRCN) at The University of Tokyo Institutes for Advanced Study (UTIAS), Tokyo, Japan

**Keywords:** Personal recovery, Culture, Qualitative research, Japan, Mental illness

## Abstract

**Background:**

The conceptualization of personal recovery began in Europe and North America and has spread worldwide. However, the concept of personal recovery in addition to recovery-promoting factors may be influenced by culture. We explored how users of mental health services in Japan perceive their own personal recovery and the factors that promote it.

**Methods:**

We conducted semi-structured interviews and focus group interviews with individuals using mental health services. The interview data were analysed using thematic analysis with a grouped framework analysis approach. We used a coding framework based on the existing CHIME framework (connectedness, hope and optimism about the future, identity, meaning in life, and empowerment).

**Results:**

Data were obtained from 30 users of mental health services (mean age: 40.4 years; 46.7% women; 50.0% with schizophrenia). “Compassion for others” was newly extracted in “Connectedness”, and “Rebuilding/redefining identity not being as shaped by social norms” was newly extracted in “Identity” as personal recovery. “Positive experiences in childhood” (including positive parenting support from neighbours) was newly extracted as a recovery-promoting factor.

**Conclusions:**

Our unique findings on the rebuilding identity/defining identity free from conformity to social norms due to interactions with familiar people, including peers, may be culture dependent. This study raises overarching questions regarding how socio-cultural values influence the development of identity and personal values and how they are in turn reflected in personal recovery.

**Supplementary Information:**

The online version contains supplementary material available at 10.1186/s12888-022-03750-4.

## Introduction

Personal recovery is a unique process that involves changing one’s attitudes, values, feelings, goals, skills, and/or role and developing new meaning in life beyond illness [1]. Based on their systematic review of the literature, Leamy et al. [2] described a conceptual framework of personal recovery that includes the following elements: connectedness; hope and optimism about the future; identity; meaning in life; and empowerment (CHIME) [2]. The CHIME recovery framework led to the development of a questionnaire about the process of recovery (QPR) [3, 4], which was later translated into Chinese [5], Swedish [6], and Japanese [7].

Regarding the recovery-promoting factors, multiple items have been identified in qualitative studies and literature reviews. For example, one systematic review indicated that recovery-promoting factors included (1) adjustment, coping, and reappraisal; (2) responding to the illness; and (3) social support, close relationships, and belonging [8]. Another systematic review identified five factors: (1) social support, (2) faith and spirituality, (3) personal agency and hope, (4) environmental resources, and (5) positive support and holistic care from services [9].

An individual’s culture adds another dimension to the concept of personal recovery and recovery-promoting factors. For example, a systematic literature review of Nordic research on personal recovery indicated the existence of a need to identify the process of recovery that reflected the Nordic mental health care systems [10]. Several studies have discussed cultural differences in the conceptualization of personal recovery [11–13]. Specifically, one literature review showed that people from Black and minority ethnic backgrounds emphasized spirituality and stigma in their recovery, and it also identified themes such as cultural facilitating factors and collectivist notions of recovery [2]. The available literature is limited on the conceptualization of personal recovery and the factors that promote it in Asian countries, including Japan. A review of Asian perspectives on personal recovery in mental health showed that support from family, friends, and social connections was the most salient recovery-promoting factor, while religious stigma (e.g., the concept of karma led to mental illnesses being viewed as punishment for prior bad deeds), discrimination, gendered norms, and negative societal perceptions of mental illness hindered recovery [14].

Understanding culture-specific concepts of personal recovery and recovery-promoting factors is essential for recovery-oriented support because it can help researchers and clinicians identify the areas that need to be assessed and focused on for care. However, little is known regarding these conceptualizations in Japan. In this study, we aimed to explore (1) the concept of personal recovery and (2) factors that promote recovery among users of mental health services in Japan. The CHIME framework is the most comprehensive description of the recovery process, with each domain within the framework containing a specific set of dimensions (Additional file 1: Table S1). CHIME has been applied in numerous Anglophone countries [12]. A recent scoping review of personal recovery conceptualisations [15] supports CHIME as a widely endorsed framework but recommends that it be adapted to the cultural specifics of the populations to which it is applied. The CHIME framework has been used internationally and to identify cultural differences in recovery concepts [12, 16]. An overlap exists between the constructs and facilitators of personal recovery. For example, Wood and Alsawy [9] consider social support, hope, and spirituality as recovery facilitators, while Leamy et al. [2] view them as elements of the concept of recovery. In essence, these factors can be considered as both recovery facilitators and as part of the recovery process. In our study, we first evaluated whether the CHIME framework could be replicated for international comparison. We then analysed whether the CHIME framework could be replicated with regard to the recovery of people living in Japan or whether cultural differences precluded it. In order to apply the results of this study to clinical practice, we also examined recovery facilitators to clarify not only the process of recovery but also the factors that promote recovery.

## Methods

### Study designs

In this study, we conducted qualitative research interviews to focus on the subjective perspectives of people with mental illness [17, 18]. The study involved the use of the semi-structured interviews and focus group interviews based on people’s experience of the process of recovery. Individual interviews offer rich insight into the experiences and perspectives of the participants, and focus group interviews generate data through interaction between participants [19, 20]. Individual and focus group interviews were used because having multiple data sources in qualitative research improves the reliability of the results and enables triangulation that enriches the quality of the information [21–23].

### Participants

We conducted interviews with users of mental health services in Japan. Inclusion criteria were as follows: (1) age ≥ 16 years, (2) ability to participate and give informed consent, and (3) use of mental health services. For study participants under the age of 20, informed assent was obtained from the participants and written informed consent was obtained from their parents. We included participants with any mental health difficulties because the concept of personal recovery involves a person who has experienced mental health difficulties and it tends to be transdiagnostic. In addition, previous studies on personal recovery did not limit the diagnosis [2, 4]. In our survey, the participants self-reported their diagnosis from a list of diagnostic categories, which included the categories ‘other’ and ‘not known’. We continued data collection until reaching theoretical saturation, which is defined as the point at which researchers have gathered enough data such that more sampling will not provide more information related to their research questions [24].

The present study used purposive sampling to get a deeper understanding of participants’ experiences [24]. We recruited the participants from five community-based mental health services in urban, suburban, and rural communities in Japan. In the first step of the recruitment process, mental health staff talked to service users about the study and showed them flyers that explained its aims and methods. Next, we explained the details of the study to interested individuals face to face or by phone, and those who agreed to participate provided written informed consent face to face. Each potential participant was informed of the purpose, methods, and funding of the study; researcher affiliations and conflicts of interest; the anticipated benefits and potential risks of the study; and the discomfort it might cause. The potential participants were informed of their right to refuse to participate in the study or to withdraw their consent to participate at any time without reprisal. Participation was voluntary, and participants had the option to discontinue if they felt distressed or otherwise did not wish to continue with the study. We promised to protect the privacy and confidentiality of the participants’ personal information. They were informed that their information would be presented anonymously. No one refused to participate or dropped out.

### Data collection

After reviewing the relevant literature on qualitative interviews, our research team developed the interview guide. We tested a pilot interview guide to ensure the feasibility of the interview and discussed findings with experts in qualitative research. We made appropriate modifications to enhance the credibility of the findings and created a semi-structured interview guide. By referring to a previous study [25], we decided to ask the participants about their process of recovery. The first interview question was, “Have there been any recent changes since you experienced mental illness?” Our intent was to focus on recent positive changes, not negative changes that occurred at the time of onset of the mental health illness. Regarding the recovery-promoting factors, we asked participants to identify factors that influenced the experience and process. The interview included the following questions, with questions 4 and 5 focusing on recovery-promoting factors in particular:

1. Has there been any recent change since you experienced mental illness? If yes, please tell me what the change was.

(Our intent was to ask about any turning point of time/situation when/where the participant’s life changed considerably.)

2. Is there anything you have gained from experience? If yes, please tell me what it was.

(Our intent was to ask about any positive change or any change in values or attitudes.)

3. Please describe your recovery in a word.
4. Are there any experiences or values that influenced your process of recovery? If yes, please tell me what they were.
5. Is there anyone who has influenced your process of recovery? If yes, please tell me what role that person has in your life.

We used introductory questions, follow-up questions, probing questions, and interpreting questions to promote positive interactions and stimulate the participants to talk about their experiences [19]. The interviews were conducted by the first author (AK). AK was a female PhD student who has worked as a clinical and research social worker in mental health settings. AK had training in qualitative methods. AK was not involved in the clinical care of any of the participants. All interviews were audio-recorded and transcribed. Data were collected between June 2017 and October 2017.

We explained the characteristics of the individual and focus group interviews to the participants and asked them to select which type of interview they would like to do. The interviews (60 min) and focus group interviews (90 min) were conducted in an interview room in clinics, a hospital, and a mental health community facility. Entry into the room was restricted to the interviewer and the participants to protect the participants’ privacy. No repeat interviews (a qualitative longitudinal data collection method) were carried out.

We also collected quantitative data to indicate participants’ process of recovery, current mental well-being, and health and disability. The self-report questionnaires were the QPR [3, 7], the WHO 5 Well-being Index (WHO-5) [26–30], and the WHO Disability Assessment Schedule 2.0 (WHODAS 2.0) [31, 32], respectively.

All participants received 5000 Japanese Yen (JPY) (equivalent to about 40 EUR or US$45) for each interview. AK made field notes after the interviews. We recorded contextual information and researcher impressions to encourage researcher reflection.

We continued the data collection until we confirmed that we had sufficient data to account for all aspects of the themes. Data collection ended after we had interviewed and analysed data from 30 participants. Of these 30 people, 15 participated in an individual semi-structured interview and 15 participated in a focus group interview. None of the participants did both types of interviews; the two groups of people were separate.

### Analysis

The interviews and our analysis were conducted in Japanese, and we subsequently translated the results into English. A thematic analysis was conducted. Thematic analysis is a method for identifying and analyzing patterns (themes) within the data set related to the research questions [24].

Regarding the recovery process, we adopted a theoretical thematic analysis driven by the specific research questions [24], using a grouped framework analysis approach [33]. Framework analysis involves applying existing codes and categories to qualitative data to support answering the research questions [33]. Our coding framework was based on the existing CHIME framework [2]. This coding framework was previously used in a systematic review that described international differences in the concept of personal recovery [12]. We used the descriptions of recovery process categories from the existing CHIME framework, which was created based on research by Leamy et al. [2] (Additional file 1: Table S1) and a review by Slade et al. [12]. Across different countries, Slade et al. [12] found a similar distribution of coding for each of the five CHIME recovery processes in English publications. They stated that while the conceptual framework was valid, conceptualizing recovery using a broader research design that is applicable to other cultures is a research priority. A recent scoping review of systematic reviews and meta-analyses on the conceptualization of personal recovery showed widespread support for the CHIME conceptual framework [15]. Stuart et al. [34] conducted a review of the recovery processes of people with severe mental illness and proposed an extended version of the CHIME conceptual framework, CHIME-D, whereby D stands for difficulties. Various measures have been developed to assess the characteristics of the recovery process and outcomes. For example, Williams et al. [35] proposed assessing the validity of recovery scales based on their correlation with CHIME. The conceptual framework of CHIME has also been used in recent interview research studies and their qualitative analyses [16, 36, 37], as well as in reviews and their analyses [38, 39]. Two researchers independently engaged in the analysis for reliability [21]. First, the first author (AK) and a licensed clinical psychologist (HK) independently read the transcripts several times to familiarize themselves with the data. Second, AK and HK independently coded each line of the transcripts. Codes capture statements (units) within transcripts that seem to reflect repeated patterns of meaning [24]. Next, we charted the data into the framework matrix [33] and then entered the summarized data into the CHIME framework. Framework analysis also pays attention to the inductive approach of data related to topics that were not anticipated in advance and the revision of coding frames [33]. Thus, when units of meaning were not adequately captured by CHIME, additional frameworks or categories were identified, and data-driven thematic analysis was used.

Regarding the recovery-promoting factors, we adopted inductive thematic analysis that was driven by the data [24]. Two researchers (AK and HK) independently read the transcripts several times and coded each line of the transcripts. Finally, the two researchers identified themes among the different units. We defined the name of themes.

In addition, we summarized selected narratives from the interviews [24]. The two researchers compared their analyses with each other, discussed overlaps and differences, and resolved ambiguities in interpretation by consensus [24]. Our research team held regular discussions. The research team included clinicians (psychiatrists, psychologists, and an occupational therapist) and researchers (qualitative study, nursing, and public health). Throughout the analysis, cross-checking and discussion with research team members were utilized to validate that the themes were meaningful and to ensure that the data analysis was reliable [24]. The research team then agreed on a conceptual framework, or final coding framework. All authors reviewed the data and checked themes and conclusions. The discussions within the research team minimized bias, detected omissions, and ensured reliability [24]. We report some of the quotations with participant numbers to indicate that different participants are being quoted or to indicate that a participant is being quoted more than once.

We used Microsoft Excel to manage the qualitative data. Due to time and capacity reasons, we could not return the transcripts to the participants and ask them to provide feedback on the findings.

All methods were conducted in compliance with the World Medical Association Declaration of Helsinki. The Ethics Committee of the Faculty of Medicine, The University of Tokyo approved this study [approval No. 11506].

## Results

### Participant characteristics

The demographic characteristics of the 30 participants (mean age: 40.4 years; 46.7% women; 50.0% with schizophrenia) are shown in Table 1. Participants were mostly single or never married (73.3%), lived with their families (60.0%), and had experienced psychiatric hospitalization (60.0%). The average interview time was 48.3 min for the interviews and 65.5 min for the focus group interviews.

**Table 1:** Demographic characteristics of the participants (*N* = 30)

	*N* (mean)	% (SD)^a^
Age, years	(40.4)	(12.9)
Gender		
Male	16	53.3
Female	14	46.7
Classification of mental disorder		
Schizophrenia	15	50.0
Mood disorders	8	26.7
Other	7	23.3
Living situation		
Single	8	26.7
With family	18	60.0
Other	4	13.3
Years of education		
Less than high school	3	10.0
High school	13	43.3
University	14	46.7
Marital status		
Single/never married	22	73.3
Married	6	20.0
Other	2	6.7
Employment/studying		
Studying	2	6.7
Employed (paying job)	13	43.3
Other	15	50.0
Service use		
Medications/ therapy	12	40.0
Day care service	9	30.0
Employment support service	5	16.7
Other	4	13.3
History of psychiatric hospitalization		
0	12	40.0
≥ 1	18	60.0
Questionnaires (self-report)		
QPR-J^b^	(65.5)	(10.2)
WHO-5-J^c^	(15.9)	(3.3)
WHODAS II-J^d^	(16.3)	(12.1)

^a^ *SD* standard deviation

^b^ *QPR-J* The Japanese version of the Questionnaire about the Process of Recovery

^c^ *WHO-5-J* The Japanese version of the WHO-Five Well-Being Index

^d^ *WHODAS II-J* The Japanese version of the World Health Organization Disability Assessment Schedule II

We present our results in terms of the concept of personal recovery and the recovery-promoting factors.

#### Concept of personal recovery

No new framework that was not already adequately captured by CHIME was extracted from the data; however, new categories that fell within the existing framework were extracted. “Compassion for others” was newly extracted in “Connectedness”, and “Rebuilding/redefining identity not being as shaped by social norms” was newly extracted in “Identity”. (Additional file 2: Table S2).

### Connectedness

Connectedness was the predominantly coded theme. Participants reported experiences that fit the concept of connectedness as proposed by the CHIME recovery framework. These included peer support and support groups, relationships, support from others, and compassion for others in connectedness.

*I had a self-negative image and suicidal ideation, but after meeting people with the same experience, I thought that I could live for the future (participant 013).*

*I realized that no one could live alone, so I started to think that it’s important to connect with others and care for others (participant 006).*

*Parents said, “It’s okay if you’re in a low state. It is important to be stable” (participant 003).*

Compassion for others was newly extracted in connectedness. Through the experience of mental illness and difficulties, the participants described that they were able to imagine others’ circumstances, including difficult situations, and to accept people with different values. A greater ability to have compassion with others was identified.

*I became kinder and had more compassion for others than I did before I experienced illness (participant 021).*

*I became able to imagine the background of others and accept diversity (participant 025).*

*I want to understand the feelings of people suffering from difficulties and want to use that experience in my work (participant 028).*

I used to believe that mental illness was just being lazy, but through my own experience, I understood that mental illness was not laziness but illness, and I found it painful. I could be generous to myself and others (participant 008).

### Hope and optimism about the future

Participants talked of their hope and optimism about the future including motivation to change, belief in possibility of recovery, positive thinking, and valuing success, as well as having dreams, aspirations, and hope-inspiring relationships.

*I kept hoping to recover (participant 009).*

*I couldn’t express myself or act actively. I don’t want to adapt to society’s expectations but started to want to get closer to my ideals (participant 019).*

*I didn’t understand the meaning or purpose of living other than work. Now I’ve come to think that my presence is the happiness of the people around me. I became grateful to each other and wanted to spend more time with my family (participant 028).*

*I have a feeling of denying myself, but I began to wonder what I could do (participant 019).*

*I feel that I am not living my life, and I thought that the only choice was suicide. I had more opportunities to express myself in the recovery program and became more motivated to live with what I wanted to do (participant 019).*

### Identity

Participants reported on their experiences with identity reconstruction. These experiences included rebuilding/redefining positive sense of self, overcoming stigma, and rebuilding/redefining an identity not shaped by social norms.

*I had the prejudice that “people with mental illness are hospitalized and cannot live their lives normally. I read a book about mental illness, talked to a medical practitioner, and lived with someone with a mental illness, and that prejudice was corrected (participant 021).*

*I thought that mental illness was scary and unfamiliar. I understand that mental illness can be scientifically explained (participant 025).*

Rebuilding/redefining identity not shaped by social norms was newly extracted in identity. Participants who experienced mental illness and difficulties, moved away from the social norms that value academic success, diligence, and productivity. They redefined their identity as being less shaped by the expectations of social norms.

*I felt that I suffered from illness because of an overemphasis on educational qualifications. Treatment liberated the thought (participant 001).*

*I started to think that errors and unpredictable things are interesting (participant 012).*

*I did not doubt that hard work, good grades, and getting a good job are necessary for wonderful life. But I had no friends. I had the experience of illness and made many friends (participant 011).*

*I used to work hard and worked to the limit. Right now, I am consciously resting and not working too hard (participant 014).*

*I used to lose myself in a place where productivity was the top priority, but now I have a place to play my role (participant 018).*

*I come to think that I want to stay as I am (participant 011).*

### Meaning in life

As reported in this study, the participants’ narratives included the meaning of mental illness experiences, spirituality, quality of life, meaningful life and social roles, meaningful social and life goals, and rebuilding of life.

*I feel that many setbacks have led to my growth (participant 002).*

*I have been given the illness from God and have been working in society. Everything has gone ahead as God planned. I think it was good (participant 015).*

*I’m not impatient to cure illness, and I can focus on enjoying the present (participant 028).*

*I find life worth living in my work and other activities (participant 007).*

*I’m looking for a job to be a "working mother" for my child (participant 020).*

*I was able to learn to accept and objectively feel painful feelings with mindfulness. I came to use it in my daily life (participant 008).*

### Empowerment

Participants reported experiences that fit the concept of empowerment as proposed by the CHIME recovery framework. These included personal responsibility, control over life, and focusing upon strengths.

I used to work hard and worked to the limit. Right now, I am consciously resting and not working too hard *(participant 014)*.

I was able to notice my feelings when I was feeling unwell. I was able to accept the advice of others *(participant 003)*.

Some people accepted what I expressed in daycare *(participant 001)*.

#### Recovery-promoting factors

Three themes were identified as recovery-promoting factors, including (1) support from others, (2) recovery-oriented practices, (3) positive experiences in childhood.

##### Support from others

Communication with peers who also experienced mental illness was recovery-promoting factor. Such communication included interactions that promoted rebuilding an identity that was not shaped by social norms, or helped deconstruct social norms.

*When I felt sad that I had an illness I never wanted to have, a person who had also experienced a mental illness gave me a warm smile and warm comments (participant 011).*

*I was career-oriented, but a peer taught me to enjoy everyday life (participant 011).*

Support from family members such as unconditional positive regard and caring communication were extracted as recovery-promoting factors.

*My father didn’t understand mental difficulty. After I was diagnosed, my father’s attitude became kind and he accepted me unconditionally (participant 017).*

*My family remained calm even though I was confused and emotional, and had been with me for a long time, even when my condition was severe. It gave me a sense of security that my family wouldn’t abandon me (participant 007).*

*Parents said, "It’s okay if you’re in a low state. It is important to be stable." (participant 003).*

Respectful communication from work colleagues or friends were extracted.

*A colleague also understood my illness and treated me kindly (participant 023).*

*A colleague who has changed my values about my ideal life and living promoted recovery (participant 001).*

##### Recovery-oriented practices

Recovery-oriented practices were identified as recovery-promoting factors. These factors include person-centred care that respects the self-determination of service users. The professionals’ hopeful and recovery-oriented attitude towards the service users’ recovery led the service users from despair to hope and empowered them to recover.

*My parents only disapproved of me, but the counsellor accepted me unconditionally (participant 024).*

*I was able to talk to my supporter about my daily life. She did not limit my enjoyment and supported me (participant 021).*

*I was relieved that my doctor told me that the illness would be cured (participant 029).*

##### Positive childhood experiences

The existence of positive experiences in childhood (including positive support from neighbours) was newly extracted as a recovery-promoting factor.

*My parents raised me to believe in me. I have accepted it and have lived. That encourages recovery (participant 006).*

*My neighbour has helped me since my childhood. After I had a mental illness, the neighbour has helped me with housing and working (participant 010).*

To summarize, support from peers, family members, and work colleagues that helped redefine identity, recovery-oriented practices that brought hope and optimism about the future, and positive childhood experiences that helped build resilience were extracted as themes of recovery-promoting factors.

## Discussion

To identify culture-dependent and universal constructs and factors promoting the process of personal recovery, we conducted individual and focus group interviews with users of mental health services in Japan. We then undertook a thematic analysis of the interview data. Most of the constructs in the CHIME framework were replicated. In addition, we obtained unique findings with regard to individuals rebuilding an identity free from conformity to social norms through communications with familiar people, including peers.

### Concept of personal recovery

Our findings revealed that the CHIME personal recovery concept is generally relevant to people living in Japan. “Compassion for others” was newly extracted in “Connectedness”, and “Rebuilding/redefining identity not being as shaped by social norms” was newly extracted in “Identity”. Connectedness was the most frequently coded category in our study.

The theme of “compassion for others” was not described in the original CHIME framework and was newly extracted in our study. Our finding is compatible with a recent study by Slade et al. [40] regarding post-traumatic growth for people with psychosis and other severe mental health problems. Through experiencing the frustration, suffering, and pain associated with their mental disorders, the service users in our study were able to think of others’ emotions, thoughts, and backgrounds; accept a wide range of values; and have compassion for others. The painful experience of having a diagnosis of an often stigmatized mental illness and the associated social disadvantages may have made them feel compassionate toward others, especially those with a minority status.

In a 33-nation study that revealed differences between a “tight culture” (with strong norms and low tolerance of deviant behaviour) and a “loose culture” (with weak norms and a high tolerance of deviant behaviour), Japan was identified as a relatively tight culture [41]. In our study, participants who had social norms that valued “hard work, good grades, and getting a good job” reported encountering people with different values. By becoming acquainted with these different values, the participants relaxed their adherence to the social norms that they originally followed and came to value having friends and enjoying everyday life. During the recovery process, the experience of encountering peers and peer support workers who were not bound by the same social norms as participants enabled the participants to subsequently form identities that did not depend on their previous social norms.

### Recovery-promoting factors

Previous studies indicated that recovery-promoting factors include close relationships, social support, and positive support from mental health services [8, 9]. In addition to those themes, the theme of positive childhood experiences was extracted in our study.

Previous studies indicated that recovery-promoting factors include close relationships, social support, and positive support from mental health services [8, 9]. In our study, in addition to support and recovery-oriented practices, which were also found in previous studies, having positive childhood experiences was extracted as an original recovery-promoting factor in our study.

Support from peers with similar experiences contributed to personal recovery in ways such as reconstructing experiences, rebuilding relationships with others, and finding meaning in life. A previous review showed that peer support workers could foster hope and belief in the possibility of recovery, including empowerment, increased self-esteem, self-efficacy, self-management, social inclusion, engagement, and increased social networks [42]. Our results also indicated that communications with peers facilitated both rebuilding an identity that was not as shaped by social norms and helping to deconstruct social norms.

Recovery-oriented practices appeared as one of the recovery-promoting factors. A previous study showed that providers’ respectful communication was associated with personal recovery from mental health problems [43]. Recovery-oriented practices, including person-centred care that respects the self-determination of service users and providers’ respectful communication, may play a key role in facilitating personal recovery through avenues such as empowerment and hope/confidence.

Positive childhood experiences were extracted as an original recovery-promoting factor in our study. A previous study showed that positive childhood experiences might reduce the risk for adult depression and poor mental health, as well as promote adult relational health [44]. In our study, the internalization of positive childhood experiences directly or indirectly influenced the promotion of personal recovery. Previous personal recovery studies may have focused on external resources and not accounted for the human capital stored within individuals. We need to focus on aspects of human capital (skills, abilities, experience, motivation, intelligence, health, and productivity) that contribute to personal recovery and well-being [45].

### International comparisons

In the international comparative study, there were no significant differences between countries in the conceptual framework (CHIME framework), but there were differences in the coding phases [12]. The subcategory of “connectedness” was most frequently coded in the United States and the United Kingdom, and the authors argued that this reflects an emphasis on “community integration” and “social inclusion” [12]. In Japan, connectedness with familiar people such as family members, friends, and colleagues was extracted from the data, and the “being part of the community” was not coded. This difference between Japan and other countries may partly stem from insufficient community support in Japan [46]. In addition, it may also be influenced by Japanese and Asian cultures that emphasize close relationships. Many Asian cultures place value on fitting in and on harmonious interdependence with others. In a study on the development and validation of attitudes towards recovery questionnaires among Chinese people, the authors emphasized “family involvement” as one of the attitudes influencing personal recovery in the Chinese context [47]. In American culture, individuals seek to maintain their independence from others by discovering and expressing their unique inner attributes [48]. Among Japanese and Filipino populations, perceived emotional support positively predicted subjective well-being even after self-esteem was controlled for [49]. However, among Euro-Americans, perceived emotional support weakly predicted subjective well-being, and moreover, the association disappeared once self-esteem was statistically controlled for [49]. Another study indicated that individualistic values were negatively related to interpersonal relationships and subjective well-being for Japanese college students, but not for American college students [50]. The results of the previous studies are consistent with “connectedness” being the most frequently coded factor in our study.

### Strength and limitations of this study

Our analysis involved an interdisciplinary team of clinicians (psychiatrists/psychologists) and researchers (psychology/nursing/public health) to improve the reliability of data analysis. However, several limitations of this study warrant consideration. First, some of the study participants were recruited from community mental health service organizations known for excellent user-centred service. This might account for the high proportion of positive experiences during the process of recovery compared with the experiences of individuals using standard care services in Japan. Second, our study did not consider the duration or severity of the participants’ disorders. Third, this study was led by researchers and clinicians. The co-production of research (full involvement in research by people with mental illness) is warranted for future studies [51].

### Implications

The conceptualization of personal recovery and identification of the factors that promote it provide a theoretical foundation for changing attitudes to support recovery in the mental health field. In clinical settings, recovery-oriented practices are important for promoting personal recovery. Communication with familiar people, including peers who help users of mental health services free themselves from conformity to social norms, might be important for personal recovery in the Japanese culture.

Our study found that the major facilitators for the process of personal recovery include daily natural and intentional support for rebuilding identity by peers and peer support workers. The conceptual framework provides a theoretical foundation for treatments and support for mental health recovery in Japan. In addition to the existing personal recovery concept of CHIME, it is important to address “compassion for others” and “rebuilding/redefining identity not shaped by social norms”. Supporting the development of relationships with peers who have similar experiences will be an important clinical focus. This study will contribute to avoiding a monocultural perspective on personal recovery. These results have important implications for organizational change in the medically oriented and professional-led mental health service systems. Most medically oriented and professional-led treatment and support programs in Japan focus on improving the individual. However, a perspective that focuses on improving the social structure (the social norms extracted in this study) surrounding the individual might make a much greater contribution to personal recovery than an approach that focuses solely on the individual.

## Conclusion

In conclusion, our study demonstrated the constructs for personal recovery and the factors that promote it in users of mental health services in Japan. Most of the constructs in the CHIME framework were replicated and thus may be regarded as universal. In contrast, our unique findings of rebuilding identity free from conformity to social norms through communications with familiar people, including peers, may be culture dependent. This study from a East Asian country may posit more universal questions of how the development of identity and personal values [52, 53] are influenced by socio-cultural values and how they are in turn reflected in an individual’s recovery journey.

## Supplementary Information

**Additional file 1: Table S1.** The descriptions of recovery process categories by collating existing CHIME framework^a^.

**Additional file 2: Table S2.** The concept of personal recovery for mental health service users in Japan and selected narratives from the interviews.

## Data Availability

The data analysed during this study are included in this published article and its supplementary information files.

## Abbreviations

QPR: The questionnaire about the process of recovery
WHO-5: The World Health Organisation Five Well-Being Index
WHO-DAS2.0: The World Health Organization Disability Assessment Schedule 2.0

## References

[1] Anthony WA (1993). Recovery from mental illness: the guiding vision of the mental health service system in the 1990s. Psychosoc Rehabil J.

[2] Leamy M, Bird V, Le Boutillier C, Williams J, Slade M (2011). Conceptual framework for personal recovery in mental health: systematic review and narrative synthesis. Br J Psychiatry.

[3] Neil S, Pitt L, Kilbride M, Welford M, Nothard S, Sellwood W (2009). The questionnaire about the process of recovery (QPR): a measurement tool developed in collaboration with service users. Psychosis..

[4] Shanks V, Williams J, Leamy M, Bird VJ, Le Boutillier C, Slade M (2013). Measures of personal recovery: a systematic review. Psychiatr Serv.

[5] Chien WT, Chan ZC (2013). Chinese translation and validation of the questionnaire on the process of recovery in schizophrenia and other psychotic disorders. Res Nurs Health.

[6] Argentzell E, Hultqvist J, Neil S, Eklund M (2017). Measuring personal recovery—psychometric properties of the Swedish questionnaire about the process of recovery (QPR-Swe). Nord J Psychiatry.

[7] Kanehara A, Kotake R, Miyamoto Y, Kumakura Y, Morita K, Ishiura T (2017). The Japanese version of the questionnaire about the process of recovery: development and validity and reliability testing. BMC Psychiatry..

[8] Kanehara A, Kotake R, Miyamoto Y, Kumakura Y, Morita K, Ishiura T (2020). Correction to: The Japanese version of the questionnaire about the process of recovery: development and validity and reliability testing. BMC Psychiatry..

[9] Wood L, Alsawy S (2018). Recovery in psychosis from a service user perspective: a systematic review and thematic synthesis of current qualitative evidence. Community Ment Health J.

[10] Schön UK, Rosenberg D (2013). Transplanting recovery: research and practice in the Nordic countries. J Ment Health.

[11] Tse S, Davidson L, Chung KF, Ng KL, Yu CH (2014). Differences and similarities between functional and personal recovery in an Asian population: a cluster analytic approach. Psychiatry..

[12] Slade M, Leamy M, Bacon F, Janosik M, Le Boutillier C, Williams J (2012). International differences in understanding recovery: systematic review. Epidemiol Psychiatr Sci.

[13] Slade M, Amering M, Farkas M, Hamilton B, O’Hagan M, Panther G (2014). Uses and abuses of recovery: implementing recovery-oriented practices in mental health systems. World Psychiatry.

[14] Kuek JHL, Raeburn T, Wand T (2020). Asian perspectives on personal recovery in mental health: a scoping review. J Ment Health..

[15] van Weeghel J, van Zelst C, Boertien D, Hasson-Ohayon I (2019). Conceptualizations, assessments, and implications of personal recovery in mental illness: a scoping review of systematic reviews and meta-analyses. Psychiatr Rehabil J.

[16] Brijnath B (2015). Applying the CHIME recovery framework in two culturally diverse Australian communities: qualitative results. Int J Soc Psychiatry.

[17] Tong A, Sainsbury P, Craig J (2007). Consolidated criteria for reporting qualitative research (COREQ): a 32-item checklist for interviews and focus groups. Int J Qual Health Care.

[18] Flick U (2007). Designing qualitative research.

[19] Kvale S (2007). Doing interviews.

[20] Barbour R (2007). Doing focus groups.

[21] Gibbs GR (2007). Analyzing Qualitative Data.

[22] Flick U (2007). Managing quality in qualitative research.

[23] Lambert SD, Loiselle CG (2008). Combining individual interviews and focus groups to enhance data richness. J Adv Nurs.

[24] Liamputtong P (2017). Research methods in health: foundations for evidence-based practice.

[25] Ajayi S, Billsborough J, Bowyer T, Brown P, Hicks A, Larsen J, et al. Getting back into the world: reflections on lived experiences of recovery. Rethink recovery series: volume 2, https://www.basw.co.uk/system/files/resources/basw_94545-8_0.pdf. Accessed 9 January 2022.

[26] The Psychiatric Research Unit at the Mental Health Centre North Zealand. The WHO-5 website. About the WHO-5. https://www.psykiatri-regionh.dk/who-5/Pages/default.aspx. Accessed 9 January 2022.

[27] Staehr JK. The use of well-being measures in primary health care—the Dep-care project. In: World Health Organization, regional Office for Europe: well-being measures in primary health care – the DepCare project. Geneva: World Health Organization. https://www.euro.who.int/__data/assets/pdf_file/0016/130750/E60246.pdf. Accessed 9 January 2022.

[28] The Psychiatric Research Unit at the Mental Health Centre North Zealand. The WHO-5 website. WHO-5 Questionnaires. WHO5 – Japanese, https://www.psykiatri-regionh.dk/who-5/Documents/WHO5_Japanese.pdf. Accessed 9 Jan 2022.

[29] Awata S, Bech P, Yoshida S, Hirai M, Suzuki S, Yamashita M (2007). Reliability and validity of the Japanese version of the World Health Organization-five well-being index in the context of detecting depression in diabetic patients. Psychiatry Clin Neurosci.

[30] Awata S, Bech P, Koizumi Y, Seki T, Kuriyama S, Hozawa A (2007). Validity and utility of the Japanese version of the WHO five well-being index in the context of detecting suicidal ideation in elderly community residents. Int Psychogeriatr.

[31] Üstün TB, Kostanjsek N, Chatterji S, Rehm J, eds. Measuring health and disability: manual for WHO disability assessment schedule. WHODAS 2.0. Geneva: World Health Organization; 2010, https://apps.who.int/iris/handle/10665/43974. Accessed 9 Jan 2022.

[32] World Health Organization, translated by M Tazaki, T Yamaguchi, Y Nakane. Measuring Health and Disability: Manual for WHO Disability Assessment Schedule WHODAS 2.0, https://www.nippyo.co.jp/shop/author/4109.html. Accessed 9 Jan 2022.

[33] Gale NK, Heath G, Cameron E, Rashid S, Redwood S (2013). Using the framework method for the analysis of qualitative data in multi-disciplinary health research. BMC Med Res Methodol.

[34] Stuart SR, Tansey L, Quayle E (2017). What we talk about when we talk about recovery: a systematic review and best-fit framework synthesis of qualitative literature. J Ment Health.

[35] Williams J, Leamy M, Bird V, Harding C, Larsen J, Le Boutillier C, Oades L, Slade M (2012). Measures of the recovery orientation of mental health services: systematic review. Soc Psychiatry Psychiatr Epidemiol.

[36] Piat M, Seida K (2018). Supported housing for persons with serious mental illness and personal recovery: what do families think?. Int J Soc Psychiatry.

[37] Apostolopoulou A, Stylianidis S, Issari P, Chondros P, Alexiadou A, Belekou P, Giannou C, Karali EK, Foi V, Tzaferou F (2020). Experiences of recovery in EPAPSY’s community residential facilities and the five CHIME concepts: a qualitative inquiry. Front Psychiatry.

[38] Wetzler S, Hackmann C, Peryer G, Clayman K, Friedman D, Saffran K, Silver J, Swarbrick M, Magill E, van Furth EF, Pike KM (2020). A framework to conceptualize personal recovery from eating disorders: a systematic review and qualitative meta-synthesis of perspectives from individuals with lived experience. Int J Eat Disord.

[39] Jagfeld G, Lobban F, Marshall P, Jones SH (2021). Personal recovery in bipolar disorder: systematic review and "best fit" framework synthesis of qualitative evidence - a POETIC adaptation of CHIME. J Affect Disord.

[40] Slade M, Rennick-Egglestone S, Blackie L, Llewellyn-Beardsley J, Franklin D, Hui A (2019). Post-traumatic growth in mental health recovery: qualitative study of narratives. BMJ Open.

[41] Gelfand MJ, Raver JL, Nishii L, Leslie LM, Lun J, Lim BC (2011). Differences between tight and loose cultures: a 33-nation study. Science..

[42] Repper J, Carter T (2011). A review of the literature on peer support in mental health services. J Ment Health.

[43] Wong EC, Collins RL, Breslau J, Burnam MA, Cefalu MS, Roth E (2019). Associations between provider communication and personal recovery outcomes. BMC Psychiatry.

[44] Bethell C, Jones J, Gombojav N, Linkenbach J, Sege R (2019). Positive childhood experiences and adult mental and relational health in a statewide sample: associations across adverse childhood experiences levels. JAMA Pediatr.

[45] Slade M, Oades L, Jarden A (2017). Wellbeing, recovery and mental health.

[46] Kasai K, Ando S, Kanehara A, Kumakura Y, Kondo S, Fukuda M (2017). Strengthening community mental health services in Japan. Lancet Psychiatry.

[47] Mak WWS, Chan RCH, Yau SSW (2018). Development and validation of attitudes towards recovery questionnaire across Chinese people in recovery, their family carers, and service providers in Hong Kong. Psychiatry Res.

[48] Markus HR, Kitayama S (1991). Culture and the self: implications for cognition, emotion, and motivation. Psychol Rev.

[49] Uchida Y, Kitayama S, Mesquita B, Reyes JA, Morling B (2008). Is perceived emotional support beneficial? Well-being and health in independent and interdependent cultures. Personal Soc Psychol Bull.

[50] Ogihara Y, Uchida Y (2014). Does individualism bring happiness? Negative effects of individualism on interpersonal relationships and happiness. Front Psychol.

[51] The best research is produced when researchers and communities work together. Nature. 2018;562(7725):7. https://www.nature.com/articles/d41586-018-06855-7. 10.1038/d41586-018-06855-7 30283119

[52] Sagiv L, Roccas S, Cieciuch J, Schwartz SH (2017). Personal values in human life. Nat Hum Behav.

[53] Kasai K, Fukuda M (2017). Science of recovery in schizophrenia research: brain and psychological substrates of personalized value. NPJ Schizophr.

